# PARP-Targeted Radiotheranostics with Auger Electrons: An Updated Overview

**DOI:** 10.3390/cimb46040190

**Published:** 2024-03-31

**Authors:** Luca Filippi, Luca Urso, Laura Evangelista

**Affiliations:** 1 Nuclear Medicine Unit, Department of Oncohaematology, Fondazione PTV Policlinico Tor Vergata University Hospital, 00133 Rome, Italy; 2 Department of Translational Medicine, University of Ferrara, 44124 Ferrara, Italy; luca.urso@unife.it; 3 Nuclear Medicine Unit, Onco-Haematology Department, University Hospital of Ferrara, 44124 Ferrara, Italy; 4 Department of Biomedical Sciences, Humanitas University, 20072 Milan, Italy; laura.evangelista@hunimed.eu; 5 IRCCS Humanitas Research Hospital, 20089 Milan, Italy

**Keywords:** Auger electrons, radionuclide therapy, prostate cancer, triple negative breast cancer, glioblastoma

## Abstract

Auger electrons (AEs) represent an intriguing topic in the field of radionuclide therapy. They are emitted by several radionuclides commonly used in nuclear medicine (indium-111, iodine-123, iodine-125), allowing for highly localized energy deposition and thus exerting a radiotoxic effect on specific cellular and sub-cellular targets. However, due to their short range in matter, AEs have had limited use in therapeutic applications so far. In recent years, the synthesis of various radiopharmaceuticals capable of binding to the enzyme poly(ADP-ribose) polymerase 1 has reignited interest in this type of therapy, laying the groundwork for a theranostic approach based on radionuclides emitting AEs. The enzyme PARP-1 operates enzymatically in close proximity to DNA that represents the prime target of radionuclide therapies. Following this trend, several PARP-targeted radiopharmaceuticals for AE-based theranostics have been developed. We provide an updated overview of preclinical studies focused on the applications of this new theranostic approach in glioblastoma, breast, prostate and ovarian carcinoma, and pancreatic adenocarcinoma.

## 1. Introduction

**Figure 1:**
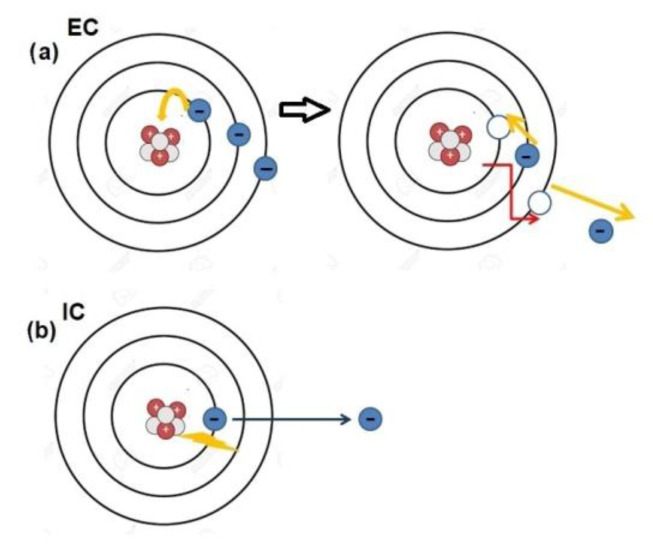
Schematic representation of Auger electron (AE) production. (**a**) In the electron-capture (EC) decay processes, vacancies are left in electron shells (K, L, M …), then filled by atomic electrons from higher energy levels (left side). In the process of falling to a lower energy shell to fill a vacancy, electron energy is lost as a photon of x-radiation that may collide with an atomic electron, resulting in the emission of an AE. (**b**) Internal conversion (IC) is an atomic decay process where an excited nucleus interacts electromagnetically with one of the orbital electrons that is knocked out from the orbit (AE). After filling the vacancy with the other orbital electron, an Auger electron can be generated in the same manner as (**a**). Illustration is authors’ own work, created with Biorender.com (accessed on 3 January 2024).

Several radionuclides commonly used in nuclear medicine, such as indium-111 (^111^In) or gallium-67 (^67^Ga), undergo decay through electron capture (EC) or internal conversion (IC) (Figure 1) [1]. This decay process results in the emission of a cascade of low-energy electrons, known as Auger electrons (AEs). AEs derive their name from the French scientist Pierre Auger, who was one of the most versatile personalities of the nineteenth century. His remarkable contributions as a physicist and educator spanned the period between the two World Wars and continued beyond. Upon relocating to Canada after the Second World War, he dedicated himself to using radio broadcasts to popularize various scientific subjects, even delving into poetry writing. He authored two volumes of dialogues between an imaginary scientist and a humanist and, notably, engaged in sculpting small cast bronze statues. Despite all of these merits, he was never awarded the Nobel Prize [2].

On par with the scientist they owe their name to, AEs represent an intriguing issue for their potential applications as a therapeutic tool in oncology. Indeed, particle-emitting radioisotopes have been routinely utilized in radionuclide therapies for many years. For instance, beta-emitters like yttrium-90 (^90^Y) or lutetium-177 (^177^Lu) have been applied for the treatment of hepatic malignancies, neuroendocrine tumors and, more recently, prostate cancer [3,4,5]. As a matter of fact, two ^177^Lu-based radiopharmaceuticals, Luthathera^TM^ and Pluvicto^TM^, have been approved by regulatory authorities for clinical use in the last several years [6,7]. Along this trajectory, some clinical trials are underway for the assessment of alpha-emitters such as actinium-225 or bismuth-213 as therapeutic agents for metastatic prostate cancer therapy, showing promising results due to their high linear energy transfer (LET) and short range in matter [8,9]. AE-based radionuclide therapies have distinct characteristics, with both advantages and disadvantages compared to beta- and alpha-emitters [10]. The majority of AEs have low energy levels (<1 KeV) and exhibit a very limited range in matter (500 nm), even more restricted than that of alpha particles (50–100 μm). When a cascade of AEs is emitted, it can result in highly localized energy deposition, causing a series of molecular damages such as DNA breaks or lipid oxidation. Hypothetically, AE-based therapy could yield potent radiobiological effects, with energy deposition occurring in specific cellular or subcellular regions, thereby minimizing off-target damage. The main advantages and disadvantages of alpha, beta, and Auger emitters are schematized in Table 1.

In past years, the radiopharmaceutical [^111^In]-DTPA-octreotide, due to its ability to selectively bind to somatostatin receptors (especially subtypes 2 and 5) expressed by neuroendocrine tumors (NET), has been widely employed in this type of neoplasia [11,12]. Consequently, given that indium-111 emits AEs, some investigations have been conducted to assess the potential of the radiopharmaceutical [^111^In]-DTPA-octreotide as a therapeutic agent in NET. Krenning et al. conducted a Phase 1 clinical trial involving 30 patients with advanced somatostatin receptor 2 (sstr-2)-positive neuroendocrine malignancies using [^111^In]-DTPA-octreotide [13]. Patients received up to 14 doses of [^111^In]-DTPA-octreotide, ranging from 6 to 7 GBq each, administered at least two weeks apart, with a maximum cumulative radioactivity of 74 GBq. Among those who received more than 20 GBq, eight patients had stable disease (SD) and six patients experienced tumor size reduction. Minimal side effects were observed up to 2 years post-treatment, primarily transient drops in platelet and lymphocyte counts. Similarly, Valkema et al. conducted a Phase 1 trial involving 50 patients with sst-2-positive tumors, administering [^111^In]-DTPA-octreotide every 2 weeks to several months for a total of 12 or more doses, ranging from 2 to 11 GBq, totaling 20–160 GBq [14]. Among 40 evaluated patients, 21 (52.5%) showed therapeutic benefit, including stable disease in 14 patients, minor remissions in 6 patients, and partial remission in 1 patient. Most patients experienced mild hematopoietic toxicity, but three out of six patients who received a total dose exceeding 100 GBq developed myelodysplastic syndrome or leukemia.

Nevertheless, despite these promising prospects, only a few clinical trials involving AE-emitting radiopharmaceuticals have been conducted, and none have been approved for clinical use by regulatory authorities [15].

However, the history of nuclear medicine has taught us that the success or failure of a nuclear medicine technique often depends on the precise pairing of the appropriate ligand with the correct radionuclide [16]. Along this trajectory, AE therapy is attracting new interest from the scientific community regarding its potential increased efficacy when using tracers directed towards poly(ADP-ribose) Polymerase 1 (PARP-1).

PARP-1 is a distinct biomarker found in various malignancies, including glioblastoma multiforme (GBM), breast, prostate, and ovarian cancer [17]. PARP enzymes play a crucial role in maintaining genomic stability by orchestrating DNA repair processes. However, in the context of cancer, particularly in breast and ovarian tumors, PARP enzymes can have a dual role, both protecting against genomic instability and promoting tumor progression [18,19]. PARP enzymes, in fact, are involved in the repair of single-strand DNA breaks (SSBs) through the base excision repair (BER) pathway. When SSBs occur, PARP enzymes are recruited to the site of damage, where they catalyze the addition of poly(ADP-ribose) chains to target proteins, facilitating the recruitment of DNA repair factors. This process helps to prevent the accumulation of DNA damage and maintains genomic stability. In cancer cells with defects in homologous recombination (HR) repair, such as those harboring BRCA1 or BRCA2 mutations, PARP inhibition leads to so-called synthetic lethality. In these cells, PARP inhibition impairs the repair of DNA double-strand breaks (DSBs), resulting in the accumulation of unrepaired lesions and subsequent cell death. This therapeutic strategy has been particularly effective in treating BRCA-mutated breast and ovarian cancers, exploiting the concept of the synthetic lethality to selectively target cancer cells while sparing normal cells. However, paradoxically, PARP inhibition can also induce genomic instability in cancer cells. Inhibition of PARP activity may lead to the persistence of DNA lesions, which can ultimately result in the generation of DSBs and chromosomal rearrangements. This phenomenon, known as “PARP trapping”, highlights the complex interplay between PARP enzymes and genomic stability in cancer cells [20]. In this scenario, targeting PARP activity can be exploited as a therapeutic strategy to induce synthetic lethality in tumors with HR repair defects. Nevertheless, the dual role of PARP inhibition in both protecting against and promoting genomic instability underscores the need for further research to optimize its therapeutic potential in cancer treatment.

The significant role of PARP-1 in cancer has been solidified by the development of numerous PARP inhibitors (PARPi) used as therapeutic agents either independently or in conjunction with other drugs [21]. PARPi, including olaparib, rucaparib, niraparib, and talazoparib, have revolutionized cancer treatment, particularly in the management of BRCA-mutated ovarian and breast cancers. Their efficacy lies in exploiting synthetic lethality, where cancer cells with defective DNA repair mechanisms are selectively targeted. These drugs have shown significant clinical benefits, including improved progression-free survival and response rates in various cancer types [22]. However, challenges exist, such as acquired resistance and toxicities like myelosuppression and gastrointestinal events. Additionally, PARP inhibitors are effective primarily in cancers with homologous recombination deficiency (HRD), leaving a gap in treatment for HR-proficient tumors. Hence, exploring alternative therapeutic approaches is imperative to address these limitations and extend benefits to a broader patient population. Additionally, identifying predictive biomarkers beyond BRCA mutations could enhance patient selection and optimize treatment outcomes [23].

**Figure 2:**
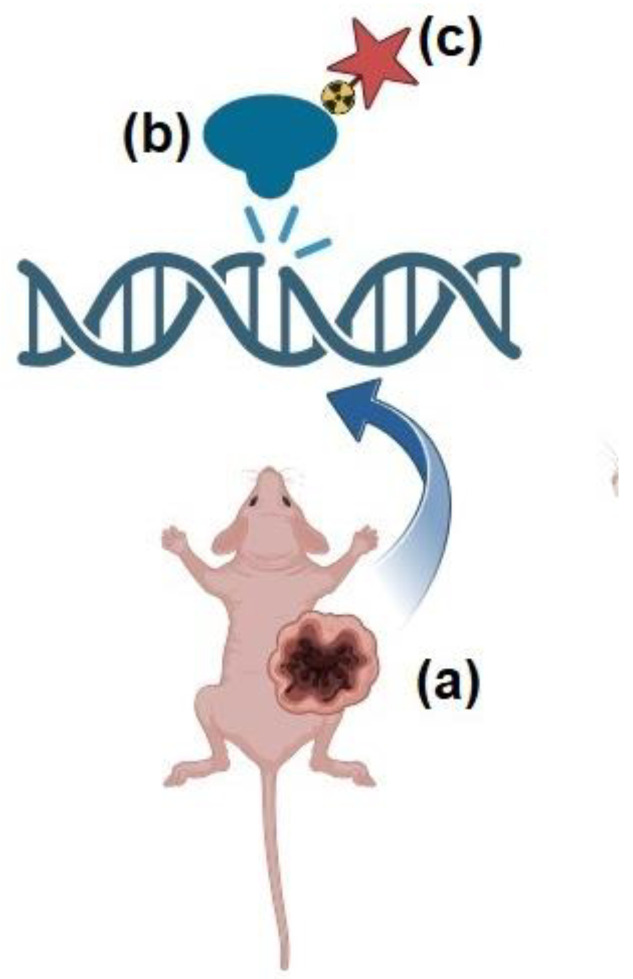
Illustration of the use of PARP-targeted radiotheranostics with Auger electrons (AEs) in preclinical studies. (**a**) depicts a mouse with a tumor exhibiting genomic instability. In such cases, poly-ADP-ribose-polymerase (PARP) functions enzymatically in close proximity to repair DNA damage (**b**). (**c**) a PARP-targeted radioligand emitting AEs can be utilized to deliver highly localized energy in close proximity to DNA (illustration is authors’ own work, created with Biorender.com, accessed on 3 January 2024.

Along this trajectory, there have been endeavors to produce PARP ligands labeled with radionuclides suitable for imaging via single photon computed emission tomography/computed tomography (SPECT/CT) or positron emission computed tomography (PET/CT) [24,25]. This aims to enhance patient selection for enrollment in PARP-targeted therapies, according to the so-called “theranostic” approach [26]. Theranostics is an innovative platform based on the administration of two molecules targeting the same tumor-associated biomarker, the first labeled with a radionuclide suitable for imaging and the second one conjugated with a radionuclide emitting particle exerting anti-tumor effects. Given that PARP-1 operates enzymatically in close proximity to the nucleus, PARP-targeted ligands have been coupled with AE-emitting radionuclides to emit a shower of electrons within a few nanometers from DNA (Figure 2). This strategy has given rise to a new paradigm of PARP-targeted Auger radio-theranostics, which has shown promising initial results in early preclinical studies (as summarized in Table 2). In the following paragraphs, we briefly cover the emerging preclinical applications of PARP-targeted AE-radiotheranostics, seeking to outline the most promising aspects and the critical issues that need to be addressed in view of clinical translation.

## 2. Glioblastoma

GBM is one of the deadliest tumors in adults, whose prognosis still remains dismal in spite of advances in diagnosis and therapy, therefore representing a compelling field of research [32]. In GBM, PARP-1 expression has been found to be a distinct biomarker of tumor biological behavior and to correlate with poor outcome. In light of the above, [^123^I]I-MAPi (Iodine-123 Meitner-Auger PARP1 inhibitor) was investigated as a possible radiotheranostic agent in GBM cells and xenograft by Pirovano et al. [27]. In the first phase of the study, U251 GBM cells were incubated with 370 kBq with [^123^I]I-MAPi and apoptosis was detected at 1 h and 24 h after incubation. In incubated cells, increased levels of apoptosis were detected at 24 h post-treatment as compared to control. In addition, the capability of [^123^I]I-MAPi to inhibit in vitro cell growth was assessed in comparison with external beam irradiation (EBIR), obtained with a cesium-137 γ-rays (662 keV) source, through colony formation assay. Cells treated with [^123^I]I-MAPi showed a steep decline in survival, confirming the high efficacy in tumor cell killing when compared to EBIR. The second phase of the study was carried out in athymic mice bearing subcutaneous patient-derived GBM xenografts, injected intratumorally with [^123^I]I-MAPi to assess tracer biodistribution via SPECT/CT. In addition, a group of mice intravenously received the PARP-inhibitor olaparib 1 h prior to [^123^I]I-MAPi for blocking assay. [^123^I]I-MAPi showed high tumor incorporation at SPECT/CT images acquired at 1, 6, and 18 h, while tracer incorporation was meaningfully reduced in mice pre-medicated with olaparib, thus supporting the specificity of the binding. Finally, the third part of the study investigated [^123^I]I-MAPi effectiveness as an anti-tumor agent in an orthotopic mouse model, bearing xenografts in the right brain hemisphere. An intratumoral single dose (0.37–1.11 MBq) of [^123^I]I-MAPi was administered to the treated cohort (*n* = 10) and the same volume of vehicle was given to the control cohort (*n* = 12). Survival data confirmed an improved survival for the [^123^I]I-MAPi treatment cohort (median survival of 58 days vs. 40 days), with no relevant side effects.

Notably, one of the most critical aspects for potential translational applications of [^123^I]I-MAPi in GBM is the route of administration. Systemic biodistribution, indeed, would result in a significant reduction in radiopharmaceutical availability due to hepatic first-pass metabolism. Furthermore, the distribution of the agents within the brain would be impeded by the blood–brain barrier (BBB). Conversely, intratumoral injection or intrathecal administration of [^123^I]I-MAPi, while ensuring a more favorable biodistribution, might not be easily feasible in current clinical practice. To partially address these challenges, the authors proposed convection-enhanced delivery (CED), an approach aimed at enhancing the distribution of molecules by maintaining a pressure gradient during interstitial infusion into white matter, to optimize [^123^I]I-MAPi for future clinical applications [33,34]. However, although promising, CED is still in a preclinical phase.

## 3. Breast Cancer

Genomic instability is a characteristic feature of breast cancer, particularly in patients harboring mutations in the DNA repair protein BRCA [35]. While BRCA mutation contributes to tumorigenesis, it also presents a therapeutic opportunity through the concept of synthetic lethality with PARPi-based treatments. Among breast cancer subtypes, triple-negative breast cancer (TNBC) poses significant challenges due to the absence of expression (or minimal expression) of estrogen receptor (ER) and progesterone receptor (PR) as well as an absence of human epidermal growth factor receptor-2 (HER2) overexpression, limiting treatment options to non-targeted chemotherapy. A recent study demonstrated promising results with the PARPi olaparib in chemotherapy-naive TNBC patients with homologous recombination deficiency, regardless of BRCA mutational status [36]. However, the heterogeneous and aggressive nature of TNBC renders some patients unresponsive to PARPi therapy. Therefore, there is an urgent need to improve the efficacy of PARPi-based treatment in this population. Sankaranarayanan and colleagues conducted an investigation into the potential of AE-based radiotheranostics in TNBC, aiming to address this pressing clinical need [28]. Following the intravenous administration of [^123^I]I-PARPi-01 in nude mice bearing subcutaneous xenografts of MDA-MB-231 cells, which exhibit characteristics of epithelial-to-mesenchymal transition indicative of aggressive metastatic TNBC, SPECT/CT imaging was performed at 4 h and 24 h post-injection. At 4 h, the images revealed predominant tracer uptake in the liver, gastrointestinal (GI) tract (including the small intestine, colon, and stomach), and thyroid. However, by 24 h, tracer uptake was only observed in the liver and thyroid, with tumor-to-blood and tumor-to-muscle ratios of 1.3 and 4.2, respectively. The high tumor-to-blood ratio was attributed to the increased binding to the plasma proteins (lipoproteins), as frequently observed with lipophilic drugs. The therapeutic phase of the study involved administering [^125^I]I-PARPi-01 (at a dosage of 8.15 ± 2.9 MBq/dose in four doses at 10-day intervals) to mice implanted with MDA-MB-231 tumors. While mice treated with [^125^I]I-PARPi-01 exhibited a significant delay in tumor growth compared to the control group, no significant reduction in tumor volume was observed during follow-up microPET scans with [^18^F]FDG, nor was there a notable survival benefit observed in the treated cohort compared to the controls.

Although the results reported by Sankaranarayanan and colleagues were still suboptimal, particularly regarding the survival data, they paved the way for some intriguing considerations. Firstly, the lipophilicity of the radiocompound determined a preferential excretion through the hepatobiliary route, thereby hindering the detection of any potential foci of tracer incorporation within the abdominal region. Secondly, adhering to the “theranostic” principles, the authors employed two different radioisotopes of iodine: iodine-123 for biodistribution studies due to its favorable physical characteristics (gamma-emission at 159 keV), and iodine-125 for therapy as an AE emitter [37].

## 4. Prostate Cancer

Despite significant advancements in therapeutic options such as androgen-receptor signaling inhibitors and radioligand therapy, metastatic castration-resistant prostate cancer (mCRPC) continues to carry a poor prognosis. Notably, a considerable proportion of mCRPC patients possess defects in DNA damage repair (DDR) genes [38]. Consequently, the Food and Drug Administration has recently approved the utilization of PARP inhibitors in patients with mutations affecting BRCA or other DDR genes [39].

Sreekumar and colleagues synthesized a radio-brominated Auger-emitting inhibitor ([^77^Br]WC-DZ) targeting PARP-1 and assessed its ability to induce cytotoxicity and DNA damage in prostate cancer cells [29]. In the initial phase of the study, the authors investigated the correlation between PARP-1 expression and Gleason score (GS), discovering a positive correlation wherein more aggressive tumors (higher GS) exhibited increased levels of PARP-1. Subsequently, they evaluated the cytotoxic effects of [^77^Br]WC-DZ in two distinct cell lines: PC-3, a commonly used model derived from CRPC bone metastases, and IGR-CaP1, a more recently developed model mimicking highly aggressive and metastatic prostate cancer. Notably, [^77^Br]WC-DZ exerted cytotoxic effects at a sub-nanomolar concentration in both PC-3 and IGR-CaP1, while no significant effects were observed at the same concentration for the “cold” compound WC-DZ-Br and rucaparib, a PARPi used in clinical practice. Interestingly, a comet assay performed on both PC-3 and IGR-CaP1 cells after exposure to [^77^Br]WC-DZ demonstrated an increased percentage of DNA in the tail, suggestive of radiation-induced DNA fragmentation. Since bromine-77 emission is not suitable for imaging with a gamma camera, a biodistribution study was carried out ex vivo; negligible uptake was found in blood, lung, muscle, bone, prostate, and pancreas (<0.5%ID/g), while slight uptake was registered in the kidney and spleen (<1.2%ID/g), with the highest uptake in the liver. The anti-tumor effect of [^77^Br]WC-DZ was investigated in mice bearing PC-3 and IGR-CaP1 xenografts. After a single intravenous dose of 56 MBq of [^77^Br]WC-DZ, a delay in tumor growth was observed, without signs of toxicity. In both PC-3 and IGR-CaP1 xenografts, a survival benefit was detected, although the effects were more pronounced for PC-3 xenografts. One of the most intriguing aspects of the study conducted by Sreekumar and colleagues is the use of bromine-77 instead of iodine-123. In this regard, bromine-77 appears particularly promising as an AE-emitting therapeutic agent: its longer half-life compared to that of iodine-123 (i.e., 57.036 h vs. 13.2 h) ensures more prolonged cytotoxic effects with no thyroid uptake, in spite of its relatively low efficiency as AE emitter (6–7 AE/decay) (Table 3).

## 5. Ovarian Cancer

Along the trajectory drawn by the previously mentioned paper [29], a further research study recently evaluated the potential of another radio-brominated rucaparib-derivative, namely [^77^Br]RD1, as potential AE-based therapeutic tool in ovarian cancer. Notably, the authors employed [^76^Br]RD1 as a diagnostic companion to assess biodistribution and pre-therapeutic biomarker (PARP-1) evaluation through PET/CT, leveraging physical properties of bromine-76 (t1/2 = 16.2 h, 55% β+) [30].

[^77^Br]RD1 binding was investigated in both human and murine ovarian cancer, in relation to PARP expression and BRCA mutation. Maximum specific binding (Bmax), equilibrium dissociation constant (Kd), and nonspecific binding slope (NS) were calculated for each cell line. The binding affinity, reflected by the equilibrium dissociation constant Kd, ranged from 6 to 30 nM, showing a certain variability in the examined cell lines owing to differences in expression of the various PARP isoforms. As concerns cytotoxicity, the authors found that the maximal effective concentration (EC50) was lower for positive-PARP than for negative-PARP cell lines (0.17 vs. 0.46, respectively), while it was not significantly influenced by BRCA mutation. Biodistribution and dosimetric studies were conducted in female mice after the intravenous administration of [^76^Br]RD1. PET dynamic scans were acquired for 1 h following administration. Subsequently, PET static images were obtained at 4 h, 24 h, and 48 h post-injection. In vivo PET quantification at 48 h post-injection was compared to ex vivo measurements. Time-activity curves showed biphasic blood clearance, with approximately 85 ± 10% of the compound excreted between 1 and 24 h. The tracer was physiologically incorporated into the liver, kidneys, stomach, intestines, and bladder. Dosimetric data obtained in mice were extrapolated using dedicated software (OLINDA) to simulate equivalent doses in female humans. Dosimetric analysis demonstrated that the bone marrow represented the dose-limiting organ, restricting the injected activity of [^77^Br]RD1 to approximately 110 GBq. In addition, since the agent showed significant hepatic uptake, liver toxicity might represent an additional dose-limiting factor. In this regard, applying the threshold commonly used for liver in external beam radiation therapy (i.e., 30 Gy), [^77^Br]RD1 activity should be limited to approximately 1600 GBq. Overall, the paper by Hoffman and colleagues emphasizes the value of [^76^Br]RD1 as a diagnostic companion, suitable for PET/CT imaging, for patient selection, and provisional dosimetry before AE-based therapy with [^77^Br]RD1. In this regard, PET/CT is characterized by higher spatial resolution and provides more robust quantitative calculations compared to SPECT/CT, employed in the case of ^123^I-based AE-theranostics, especially with the newly implemented long axial field-of-view scanners [40,41].

## 6. Pancreatic Cancer

A recently published paper by Chan et al. investigated the effectiveness of another radio-iodinated PARP-ligand, namely [^123^I]I-CC1, in three tumor cell lines (PSN1, U87MG, and MDA-MD-231) derived from pancreatic cancer, glioblastoma, and breast cancer, respectively [31]. The radiotoxic effect of [^123^I]I-CC1 was assessed in cell cultures of all three tumor cell lines, while biodistribution studies were carried out by dynamic SPECT/CT over 1 h after intravenous injection in mouse models subcutaneously implanted with tumor-derived xenografts. Additionally, the therapeutic efficacy of [^123^I]I-CC1 was evaluated in mice bearing xenografts. Exposure of tumor cells to [^123^I]I-CC1 resulted in increased expression of PARP1 at 24 h after a 1 h exposure, with a cytotoxic effect on tumor growth in vitro and a less pronounced effect in PSN1. Notably, neither “cold” CC1 nor the PARPi olaparib showed relevant effects on clonogenic survival. Dynamic SPECT/CT indicated that [^123^I]I-CC1 had prominent hepato-biliary clearance with a 2-phase decay. Ex vivo analysis of tissue samples obtained from mice exposed to [^123^I]I-CC1 to assess toxicity in the perspective of clinical translation did not show relevant findings in the liver, spleen, and intestine, with the exception of moderately increased hemosiderophages in the spleen and diffuse cytoplasmic pallor or rarefaction in hepatocytes. The most remarkable result obtained by Chan and colleagues was a significant inhibition of tumor growth in animals bearing PSN1 xenografts exposed to [^123^I]I-CC1 (3 MBq) compared to the group treated with “cold” CC1. Minor inhibition was observed in mice implanted with U87MG xenografts, while no significant response was obtained in MDA-MD-231 models. The results reported by Chan’s group emphasize the potential of PARP-targeted AE-based therapy for pancreatic ductal adenocarcinoma, which remains a malignancy with limited therapeutic options. In this regard, a discrepancy was noted between in vitro (less pronounced effects of [^123^I]I-CC1) and in vivo findings (significant tumor reduction with a single dose). The authors attributed the reasons for this difference to disparities in tracer uptake or DNA damage in vivo compared to cell culture, but it is worthy of further investigation in the perspective of translational applications.

## 7. Final Considerations

The initial experiences conducted with PARP-targeted AE-radiopharmaceuticals show promise. The advantage of AE-based PARP-ligand therapy would be, first of all, the ability to use radionuclide therapy in tumors with genomic instability, such as TNBC, GBM, and pancreatic adenocarcinoma, for which there are currently no clinically available radionuclide-based options with beta or alpha emitters. Furthermore, Auger therapy with PARP ligands might ensure lower toxicity, given the highly localized mechanism of action, although this latter aspect requires further investigation through translational studies. In this regard, from the analysis of available scientific data, some considerations can be made regarding AE-based therapy. AE-radiotheranostics might offer several key advantages in cancer treatment. Firstly, it provides unparalleled precision in irradiating cancerous cells due to the very short range of energy deposition of AEs. This precision ensures that the radiation targets the tumor cells with exceptional accuracy, minimizing damage to surrounding healthy tissues. Moreover, AEs exhibit a high linear energy transfer (LET) between 1 and 23 keV/μm, making them particularly effective in causing clustered damage when released, thanks to PARP-ligands, in proximity of DNA [10]. This high LET enables AEs to induce significant DNA damage, including double-strand breaks, which are vital in triggering cell death pathways such as apoptosis or mitotic catastrophe. Unlike other radiation particles such as beta particles, AEs have a limited crossfire effect, reducing the risk of unintended radiation exposure to neighboring healthy cells. This targeted approach not only enhances the efficacy of the treatment but also minimizes the potential for adverse side effects. Furthermore, certain AE-emitting radionuclides also emit internal conversion (IC) electrons, extending the therapeutic range of AE-based approaches. These IC electrons possess higher energies and ranges, allowing for a longer-range effect that can reach several millimeters beyond the primary target area.

However, several issues still need to be addressed. It remains unclear, as an example, which radionuclide, among those utilized in various experiments (i.e., ^123^I, ^125^I, ^77^Br), might be the most suitable for ensuring optimal anti-tumor activity while minimizing radiation exposure to dose-limiting organs such as the liver and bone marrow. In addition, certain limitations of AE-based radionuclide therapy, compared to therapies utilizing beta or alpha particles, should be emphasized. The short range in matter, which is only partially mitigated by coupling AE emitters with PARP-ligands, poses a significant challenge. Further consideration needs to be given to the limited availability of AE-emitting radionuclides and the potential co-emission of photons or beta particles alongside AEs [1]. In this regard, some AE emitters, such as indium-111 and iodine-125, are characterized by a photon/electron (p/e) ratio of 11.8 and 2.16, respectively, leading to implications for radiation safety, undesirable irradiation of normal tissues, and complications for radiation protection [1]. Another relevant issue deserving further investigation may involve future studies utilizing imaging probes other than radiopharmaceuticals, such as PARP-targeted fluorescent compounds, aimed at exploring the safety of AE-based radiotheranostics in animal models [42].

In this regard, compounds labeled with bromine-77 appear particularly promising, especially considering the potential for diagnostic pre-treatment PET studies with bromine-76. Additionally, the majority of PARP-targeted ligands demonstrate some degree of lipophilicity and significant excretion via the hepato-biliary route, resulting in suboptimal biodistribution for detecting and treating lesions in the abdominal region. To overcome this limitation, significant efforts are needed to optimize the chemical characteristics of various PARP-targeted radioligands to achieve a more favorable biodistribution and kinetics. In the coming years, the synergy between nuclear physics, radiopharmacy, and experimental nuclear medicine will be crucial in determining whether AE therapy will indeed rise from its ashes, akin to the Phoenix of ancient mythology.

## Figures and Tables

**Table 1:** Main physical characteristics, advantages and disadvantages of alpha, beta and Auger emitters.

Type of Radiation	Particle	Range in Matter	Advantages	Disadvantages
Alpha	2 neutrons +2 protons	20–70 µm	- High LET - Induction of direct and indirect damage - ds-DNA breaks - Short range in matter, limited “crossfire effect”	- Decay generating “recoiling daughters” - Limited availability
Beta	Electron	From ~1 mm up to 12 mm	- Induction of direct and indirect damage - Good availability - “Crossfire effect” may result in damage to multiple neighboring cancer cells	- Relatively low LET - “Crossfire” effect may result in collateral damage of healthy tissue - ss-DNA breaks
Auger	Electron	<1 µm	- High LET - Induction of direct and indirect damage - Short range in matter, limited “crossfire” effect - Highly localized energy deposition	- Need for targeted deposition in close proximity to critical cell targets - Limited availability - Potential co-emission of photons

µm: micrometer, mm: millimeter, LET: linear energy transfer; ds-DNA breaks: double-strand DNA breaks; ssDNA: single-strand DNA.

**Table 2:** Main pre-clinical applications of PARP-ligands and radionuclides emitting Auger electrons.

Author, Ref.	Country/Year	RPA (Indication)	Tumor	Comments
Pirovano et al. [27]	USA/2020	[^123^I]I-MAPi	GBM	Intratumoral injection of [^123^I]I-MAPi allowed to obtain high tumor incorporation and determined a survival benefit in animal models
Sankaranarayanan et al. [28]	Germany/2022	[^123^I]I-PARPi-01 (diagnostics)	TNBC	[^123^I]I-PARPi-01 mainly showed biodistribution in liver, thyroid, and gastrointestinal tract. [^125^I]-PARP-01 was capable of delaying tumor growth in animal models, but did not lead to a survival benefit
		[^125^I]I-PARPi-01 (therapeutics)		
Sreekumar et al. [29]	USA/2023	[^77^Br]WC-DZ	Prostate cancer	[^77^Br]WC-DZ was found to exert anti-tumor activity in cell lines. It determined delay in tumor growth and survival benefit in animal models
Hoffman et al. [30]	USA/2023	[^76^Br]RD1 (diagnostics)	Ovarian cancer	[^76^Br]RD1 resulted a reliable diagnostic companion for selection and provisional dosimetry with PET/CT [^77^Br]-RD1 was capable of binding with high affinity ovarian cancer cells expressing PARP, regardless of the BRCA mutational status.
		[^77^Br]RD1 (therapeutics)		
Chan et al. [31]	UK/2023	[^123^I]I-CC1	Breast, pancreatic cancer and GBM	[^123^I]I-CC1 exerted an anti-tumor effect in examined tumor cell lines. In animal models, it was particularly effective in delaying tumor growth, mainly in pancreatic cancer

RPA: radiopharmaceutical agent; GBM: glioblastoma multiforme; TNBC: triple negative breast cancer.

**Table 3:** Main physical characteristics of some relevant Auger-emitting radionuclides.

Radionuclide	Half-Life	Auger Emission	Advantages	Disadvantages
^123^I	13.2 h	14 AE/decay	- It can be easily produced - It allows imaging by SPECT/CT	- It entails unwanted radiation burden to thyroid *
^125^I	59.49 d	23 AE/decay	- It is an efficient AE emitter	- Its long half-life determines radiation safety issues - It does not allow imaging with gamma-camera
^77^Br	57.036 h	6–7 AE/decay	- Its long half-life may produce prolonged anti-tumor effects. - It can be coupled with [^76^Br] as PET diagnostic companion	- It has a relatively low efficiency as an AE emitter.

* However, the block of thyroid cells can reduce the irradiation.
